# A System of Medicine

**Published:** 1897-03

**Authors:** 


**A System of Medicine.** By Many Writers. Edited by THOMAS CLIFFORD ALLBUTT, M.D., F.R.S. Volume I. Pp. xxxix., 977. London: Macmillan and Co., Ltd. 1896.

It is more than usually fitting that a general summary of medicine of this kind should appear under the editorship of one who has spent so many years in the active acquisition of a very wide experience, and who now has a comparative degree of leisure for other work with more than an average degree of ability to present the results of his experience in an eminently attractive form. The introduction to the volume is written by the editor, and is a masterpiece of philosophical analysis, which must be read entire, and should not be mutilated by any quotation or insulted by criticism. It forms an excellent prelude to the articles in Division I., which are called Prolegomena, and are a special characteristic of this book as distinguished from the ordinary existing manuals on medicine. This division comprises twenty chapters, extending to nearly five hundred pages, on a variety of most useful topics, written by specialists mostly of world-wide reputation. We are tempted to give a list of these chapters and the names of the authors, but it is difficult to mention one without going through the list in rotation.

The chapter on anthropology and medicine opens up questions on which few physicians are qualified to write; none so well qualified as Dr. John Beddoe, whose work as Physician to the Bristol Royal Infirmary enabled him to combine the physician with the erudite anthropologist. His experience of disease in relation to colour must be very large, and it is interesting to note that "on the whole, however, the deposition of pigment in the skin and hair of mammals would seem to be the result of processes which connote or accompany health and vigour rather than the opposite." The fallacy of statistics is well illustrated by his analysis of the distribution of phthisis in France; we naturally infer that the greater density of population is the one and sufficient reason for the greater amount of phthisis in the recruits from Flanders, Picardy and Normandy. His explanation is as follows: "The fact is the tall, long-headed blond population is where it is by reason of its physical and moral qualities; its striving, ambitious, masterful character, which enabled it to occupy the best and most fertile parts of France, leaving the hills and heaths to the dark, short-headed Kelts." In the British Isles, personal observation enables him to assert that phthisis is not especially prevalent among blonds. The typical victim of phthisis is a tall person with blue eyes, a transparent complexion, and dark hair. As regards the best seed-bed for cancer, he finds that "the prevailing complexion, among the subjects of cancer in this country, is dark." But he adds: "Of course it does not necessarily follow that cancer is at all more prevalent among generally swarthy than among generally blond nations; nor am I aware of any evidence in favour of such a belief. On the contrary, there is some reason for supposing it to be a disease whose development is favoured by civilisation, comfort and intellectual progress; and these are on the whole most prevalent in the races whom Huxley calls Xanthochroi, although it is the swarthy individuals among them who suffer most." This point is of some interest in connection with the well-known prevalence of pigmented moles in elderly patients who are the victims of internal cancerous disease.

Dr. W. H. R. Rivers supplies a short chapter on temperament. After an outline of the four-fold division, on which there has been a long-maintained and singular unanimity among both medical and philosophical writers, another possible basis on psychological outlines is suggested. "As a working hypothesis at the present time, the customary tripartite division of mind might be accepted, and an endeavour be made to find the physical and mental traits which characterise the three types of Auguste Comte—the man of thought, the man of feeling, and the man of action."

The laws of inheritance in disease, and the medical geography of Great Britain, are followed by a chapter of eighty-four pages, by Professor John George Adami, on inflammation, in which an attempt is made to bring into order the very numerous recent researches upon the inflammatory process and to show whither they appear to tend. He concludes a very complete outline of the subject by defining inflammation "as the series of changes constituting the local manifestation of the attempt at repair of actual or referred injury to a part, or, briefly, as the local attempt at repair of actual or referred injury."

The chapter on climate in the treatment of disease is written by Dr. Hermann Weber and Dr. Michael G. Foster, and it is gratifying to note that "the qualities of energy, perseverance, and hope cannot be over-estimated in the treatment of chronic disease by climate." With these factors acting in co-operation with the world-wide variations of barometric and thermometric changes, we have an armamentarium which may well take its place side by side with, and acting in harmony with (not instead of), those agents which Dr. D. J. Leech summarises in his chapter on the principles of drug therapeutics. Of both kinds of treatment it may be said that they can "only act beneficially when they can exercise such influence on the morbid changes in tissues and organs as to restore the parts to a state compatible with systemic life. But in a large proportion of cases such restoration is impossible," and the quest for a suitable climate is as useless as that for a suitable drug.

The chapters on artificial aerotherapeutics, on balneology and hydrotherapeutics, the medical applications of electricity, massage, nursing, the hygiene of youth, and the general principles of dietetics in disease are of a much more complete and useful character than has generally been the case in essays on those subjects.

The subject of life insurance is now a very important part of medical work, and we have from Dr. E. Symes Thompson a very practical outline of what the examiner should do. We are delighted to see that he protests against the endless series of trivial questions and the over-examination to which would-be insurers are often called upon to submit.

After this excellent series of preliminary essays we come to Division II., which comprises two parts: one is on insolation, by Sir Joseph Fayrer, and the other deals with some of the various infections. Dr. Kanthack writes on the general pathology of infection; Dr. Julius Dreschfeld on enteric fever and on infective endocarditis; and Mr. Watson Cheyne on erysipelas and on septicæmia and pyæmia. The other writers, for the most part English and metropolitan, are well known in their special departments; they represent the best of English workers, but the work they represent is cosmopolitan. The influence of bacteria is paramount throughout this part of the volume: even in the comparatively simple and harmless furuncle Dr. Melsome points out that "whatever part constitutional states may play, it is certain that the occurrence of a furuncle is impossible without the intervention of *pus micro-organisms.* Staphylococcus pyogenes aureus or albus, or both together, are invariably present," and hence local antiseptic treatment is the one thing needful. "The various drugs alleged to have special healing properties in these affections are, so far as we know at the present time, quite useless." Dr. Dreschfeld's excellent article on enteric fever is based upon the demonstration that the specific cause of the disease is Eberth's bacillus, but he has to admit that antitoxin treatment finds as yet no place in its treatment, whereas in diphtheria and tetanus the antitoxin principle has been most successfully applied. The chapter on serum therapeutics shows us what can and what cannot be accomplished by this means.

The book is a mine of wealth, and a most welcome feature of it is the copious bibliography appended to the sections.

